# Bacterial Vaginosis and Sexually Transmitted Infections in an HIV-Positive Cohort

**DOI:** 10.3389/frph.2021.660672

**Published:** 2021-04-12

**Authors:** Karina Nava-Memije, Cecilia Hernández-Cortez, Verónica Ruiz-González, Claudia A. Saldaña-Juárez, Yazmín Medina-Islas, Roberto A. Dueñas-Domínguez, Ma. Guadalupe Aguilera-Arreola

**Affiliations:** ^1^Medical Bacteriology Laboratory, Microbiology Department, Instituto Politécnico Nacional–Escuela Nacional de Ciencias Biológicas, Mexico City, Mexico; ^2^Clinic Laboratory, Secretaria de Salud Pública, Clínica Especializada Condesa Iztapalapa, Mexico City, Mexico; ^3^Microbial Biochemistry Laboratory, Microbiology Department, Instituto Politécnico Nacional–Escuela Nacional de Ciencias Biológicas, Mexico City, Mexico; ^4^Specialized Laboratory, Secretaria de Salud Pública, Clínica Especializada Condesa, Mexico City, Mexico

**Keywords:** HIV, STI, bacterial vaginosis, polymicrobial infections, mycoplasma, chlamydia, diagnosis

## Abstract

The World Health Organization (WHO) and the Joint United Nations Programme on HIV and AIDS (UNAIDS) suggest that sexually transmitted infection (STI) surveillance should include other genital infections and not only human immunodeficiency virus (HIV). To monitor the concomitance of bacterial vaginosis (BV) and STIs in HIV-seropositive (HIV+) and HIV-seronegative (HIV–) patients, a prospective study was conducted in a cohort of 349 volunteers at a clinic specializing in treating STIs in Mexico City. Microbiological and molecular methods were used to detect STIs and dysbiosis in HIV+ and HIV– individuals. The prevalence of infection was higher in HIV+ (69.28%) than in HIV– (54.87%) individuals. BV was the most frequent infection in HIV+ individuals, and polymicrobial infections were 3 times more common in HIV+ individuals than in HIV– individuals (31.48 vs. 10.98%). Behaviors documented in a self-administered questionnaire included low condom use frequency in HIV+ individuals co-infected with BV or a STI. This finding highlights the importance of surveillance using routine microbiological evaluations for the correct management of genital infections in HIV+ patients because in the presence of HIV, the clinical presentations, courses, and therapeutic responses of some STIs can differ from those in patients without HIV infection.

## Introduction

Worldwide, sexually transmitted infections (STIs) due to *Chlamydia trachomatis* (Ct), *Neisseria gonorrhoeae* (Ng), and *Trichomonas vaginalis* (Tv) account for the highest disease burden in the STI epidemic and have been estimated in detail by the World Health Organization (WHO) (1). In contrast, less information is available regarding the prevalence and role of genital mycoplasmas, such as *Ureaplasma* spp. (Ur) and *Mycoplasma hominis* (Mh) in STIs (2). Bacterial vaginosis (BV) is not an STI but is the most common genital disorder that affects women of reproductive age, and its prevalence ranges worldwide between 36 and 43% (3, 4). BV is a dysbiosis characterized by a relatively low abundance of vaginal *Lactobacillus* sp., accompanied by polymicrobial anaerobic overgrowth (5–7). Recent data suggest a potentially important synergistic relationship between *G. vaginalis, Prevotella bivia*, and *Atopobium vaginae* in BV pathogenesis (8–11). The main outcomes related to BV include an increased risk of preterm delivery, pelvic inflammatory disease, and an increased risk of acquisition of STIs, including human immunodeficiency virus (HIV), human papillomavirus (HPV), and other sexually transmitted pathogens.

The WHO estimate reveals that during 2019, 2.1 million people were living with HIV in Latin America, and the high HIV prevalence rates in this region were possibly driven by the synergy between HIV and the other common STIs (12). In addition to immunosuppression and a decrease in normal vaginal microbiota, HIV infection contributes to increased susceptibility, promotes complications and modifies the response to the treatment of genital infections (3, 12, 13). Therefore, the WHO and Joint United Nations Programme on HIV and AIDS (UNAIDS) suggest that STI surveillance should include other genital infections and not only HIV; this inclusion is an irreplaceable component of HIV-AIDS monitoring systems (14, 15). Studies in HIV-positive populations not only allow the identification and differentiation of the frequency of microorganisms associated with genital infection but also help in understanding the current national landscape.

To improve the management of symptomatic individuals with presumed STIs, syndromic management was introduced in Mexico 30 years ago; this kind of clinical management is based on the identification of clinical symptoms and signs. Syndromic management uses algorithms based on groups of genital symptoms to guide STI treatment in people seeking care without the use of laboratory tests. Similarly, syndromic management has led to the improvement of the diagnosis of genital, ulcer-related, sexually transmissible bacterial pathogens (e.g., syphilis), but the approach has failed to control asymptomatic chlamydial, gonococcal or BV. Untreated asymptomatic STIs and/or BV are important, as they cause inflammatory changes in the lower female genital tract, which can increase the risk of HIV acquisition and result in reproductive complications (4, 12).

In Mexico, according to the CENSIDA Bulletin of the Ministry of Health in 2019, it is estimated that 12,000 new HIV infections occur. However, data about the incidence and prevalence of other STIs and dysbiosis are scarce. In our previous studies (2011 to 2013), we described an increase in the number of cases of *C. trachomatis* from 0.4 to 14%, mainly in asymptomatic women. *Ureaplasma* sp. was the most common bacteria, up to >10^4^ CFU/mL (17–42%). Dysbiosis due to *Candida* vulvovaginitis decreased from 18 to 10%, while the prevalence of BV and *T. vaginalis* was 19–21% and 0–0.5%, respectively (16, 17). Since generated new data will facilitate the improvement of surveillance programmes on converging epidemics of HIV, other sexually transmitted infections and bacterial vaginosis, we sought to investigate the concomitance of BV and STIs in HIV-seropositive (HIV+) women. Thus, we compared samples from HIV-infected (HIV+) and HIV-uninfected (HIV–) women collected at the specialized STI clinic in Mexico City using diagnostic tests that are generally not available to the public health system, as they are often expensive and/or geographically inaccessible.

## Materials and Methods

### Ethical Approval and the Recruitment Process

The ethics committees of the “Clínica Especializada Condesa (CEC)” and “Escuela Nacional de Ciencias Biológicas del Instituto Politécnico Nacional (ENCB-IPN) reviewed and approved the protocol (CEI-ENCB 001/2013 and CECITS0614, respectively).

Over a period of 18 months, 349 samples were taken from women between 18 and 45 years old. The samples were divided into two different groups in accordance with HIV serological status. In the first group, 82 HIV-seronegative (HIV–) participants were included, and in the second group, 267 HIV-seropositive (HIV+) participants were included. The inclusion criteria included not having consumed or applied oral or topical antibiotics or antimycotics in the vaginal area in the last month, not having used vaginal douches in the last 15 days and not being pregnant. Additionally, women who were receiving hormonal treatment or who did not complete 72 h of sexual abstinence were excluded. All the HIV+ volunteers enrolled in this research were previously registered as CEC patients to receive antiretroviral therapy (ART). However, it was not possible to follow the patients to assess adherence to treatment.

### Date and Sample Collection

Informed consent was obtained from each participant prior to her recruitment into the study, and a self-administered questionnaire was gathered from each participant. The questionnaire consisted of a short form to gather information about age, menstruation, previous pregnancies, use of vaginal douches, genital symptoms, reason for participating in this study, relationship status, parental status, sexual relations with other partners, the locations where the respondents met their sexual partners and other information relating to STDs. Other health issues were also included for example contraception and background HPV. Vaginal samples were obtained using a plastic Dacron swab, while a cytobrush was used to sample cervical cells for the detection of *N. gonorrhoeae* and *C. trachomatis*.

### Processing or Management of Cervicovaginal Samples

The presence of BV and trichomoniasis was studied in vaginal samples as previously described (16, 17). Amsel's clinical criteria was evaluated, the Nugent score was calculated and *G. vaginalis* culture on Casman plates was performed for all samples. Positivity in at least two of the criteria had to be present for a diagnosis of BV. *T. vaginalis* was diagnosed by observation on wet mount in physiological saline solution, and the slides were evaluated immediately after vaginal samples were collected. The presence of candidiasis was reported with the observation of budding yeast and/or pseudomycelium on wet mounts in physiological saline solution, as well as in culture in chromogenic medium (CHROMagar™, Paris, France). Since almost 10 to 30% of women have candida colonization without any symptoms, the presence of microbiological findings was correlated with specific symptoms such as itching, burning an increase in normal secretion volume or a change in secretion type. The Mycoplasma IST 2 gallery (bioMérieux, Inc., Marcy I'Etoile, France) was used for the culture and identification of *M. hominis* and *Ureaplasma* spp., according to the manufacturer's instructions. The diagnostic kit provided information regarding the presence or absence of *M. homini*s and *Ureaplasma* spp.; an estimate of the density of each organism (cut-off <10^4^ color-changing units by milliliter-CFU/mL).

Endocervical swabs were tested for the presence of *C. trachomatis* and *N. gonorrhoeae*. Samples were obtained from the endocervix with a cytobrush. Cervical samples were frozen in 2-sucrose phosphate medium until use. Deoxyribonucleic acid (DNA) extraction for *C. trachomatis* and *N. gonorrhoeae* detection was performed with a High Pure PCR Template Preparation Kit (Roche, Mannheim, Germany). The DNA was maintained at −20°C until use. Polymerase chain reaction (PCR) amplification for the genetic detection of both *C. trachomatis* and *N. gonorrhoeae* was performed with the method described previously by Aguilera-Arreola et al. (18). In addition, *N. gonorrhoeae* was cultured on Thayer-Martin medium selective gonococcal plates immediately after sampling and incubated for 48 h at 37°C under CO_2_ conditions.

### Statistical Analysis

Data analysis was carried out using SPSS Statistics 17.0 and Excel (IBM Software, and Microsoft Office). The χ^2^ test was used to determine the level of statistical significance for categorical variables. Statistical significance was defined as *p* <0.05 (19). Yates correction was used when the frequency of any variable was less than five, and if two, separated variables were compared. Fisher's exact test (Ç) was used if more than two variables were analyzed (20). Odds ratios (ORs) were calculated to describe the relative odds of the occurrence of STIs in both HIV+ and HIV– populations (21).

For the analysis of the presence of polymicrobial infections, the entire sample was divided into HIV– and HIV+ groups. Then both groups were subdivided into three different subgroups: those with no microbial infection (NMI) (any other infection in addition to HIV), with monomicrobial infection (MI) and with polymicrobial infections (PI).

## Results

### HIV+ Results

In 186 (69.96%) samples, at least one aetiological agent was found. In the remaining 81 (30.33%), no pathogens were detected. The most frequent affliction was BV (34.04%), followed by the high frequency of *Ureaplasma* sp. (32.58%) and *Candida* sp. (11.61%). Trichomoniasis (8.23%) and chlamydial cervicitis (1.87%) were less common. No cases of gonorrhea were detected by culture or PCR (Table 1).

**Table 1:** Statistical analysis of the frequency of genital infections in individuals.

**Etiology**		**HIV+ *n* (%)**	**HIV– *n* (%)**	**Pearson test (χ^2^, *p*-value)**	**Odds ratio (OR)**	**Confidence interval**
*Trichomonas vaginalis*	Positive	22 (8.2)	1 (1.2)	0.025^*^	7.27	0.97–54.82
	Negative	245 (91.8)	81 (98.8)			
BV	Positive	91 (34.08)	9 (10.97)	0.000^*^	4.22	2.02–8.82^♦^
	Negative	175 (65.92)	73 (89.03)			
Candidiasis	Positive	31 (11.61)	9 (10.97)	0.874	1.07	0.48–2.34
	Negative	236 (88.39)	73 (89.03)			
*Chlamydia trachomatis*	Positive	5 (1.87)	5 (6.09)	0.103∞	0.29	0.08–1.04
	Negative	262 (98.13)	77 (93.91)			
*Ureaplasma* sp.	Positive	87 (32.58)	27 (32.92)	0.953	0.98	0.58–1.67
	Negative	180 (67.42)	55 (67.08)			
*Mycoplasma hominis*	Positive	11 (4.11)	1 (1.21)	0.360∞	3.48	0.44–27.37
	Negative	256 (95.89)	81 (98.79)			
*Ureaplasma* sp. plus	Positive	38 (10.86)	2 (2.43)	0.003^*^	6.64	1.57–28.14^♦^
*Mycoplasma hominis*	Negative	229 (89.14)	80 (97.57)			

*No cases of gonorrhea were detected. Pearson's χ^2^ and OR tests were performed to evaluate the risk factors for HIV infection*.

* *Statistical significance (p < 0.05). ∞ Yates' correction was performed*.

♦ *The result is significant because it lies within the calculated confidence interval*.

### HIV– Results

At least one aetiological agent was found in 45 samples (54.87%) of the 82 samples analyzed. In the remaining 37 samples (45.12%), no pathogens were reported. The most frequent microorganism was *Ureaplasma* sp. (32.92%), followed by *Candida* spp. (Can) (10.97%). Less common were BV (10.97%), trichomoniasis (1.21%) and chlamydial cervicitis (6.09%). Any cases of gonorrhea were detected by both culture and PCR (Table 1).

### HIV– and HIV+ Comparisons

BV (0.000^*^), mixed infection by *Mycoplasma* sp. and *Ureaplasma* sp. (0.003^*^) and trichomoniasis (0.025^*^) were more common in HIV+ than in HIV– subjects (Table 1). In addition, the HIV+ group had a significantly greater risk of developing BV or mixed infection by *Mycoplasma* sp. and *Ureaplasma* sp. than the HIV– group (Table 1; ^*^p <0.05).

### Mono- and Polymicrobial Infections

**Figure 1:**
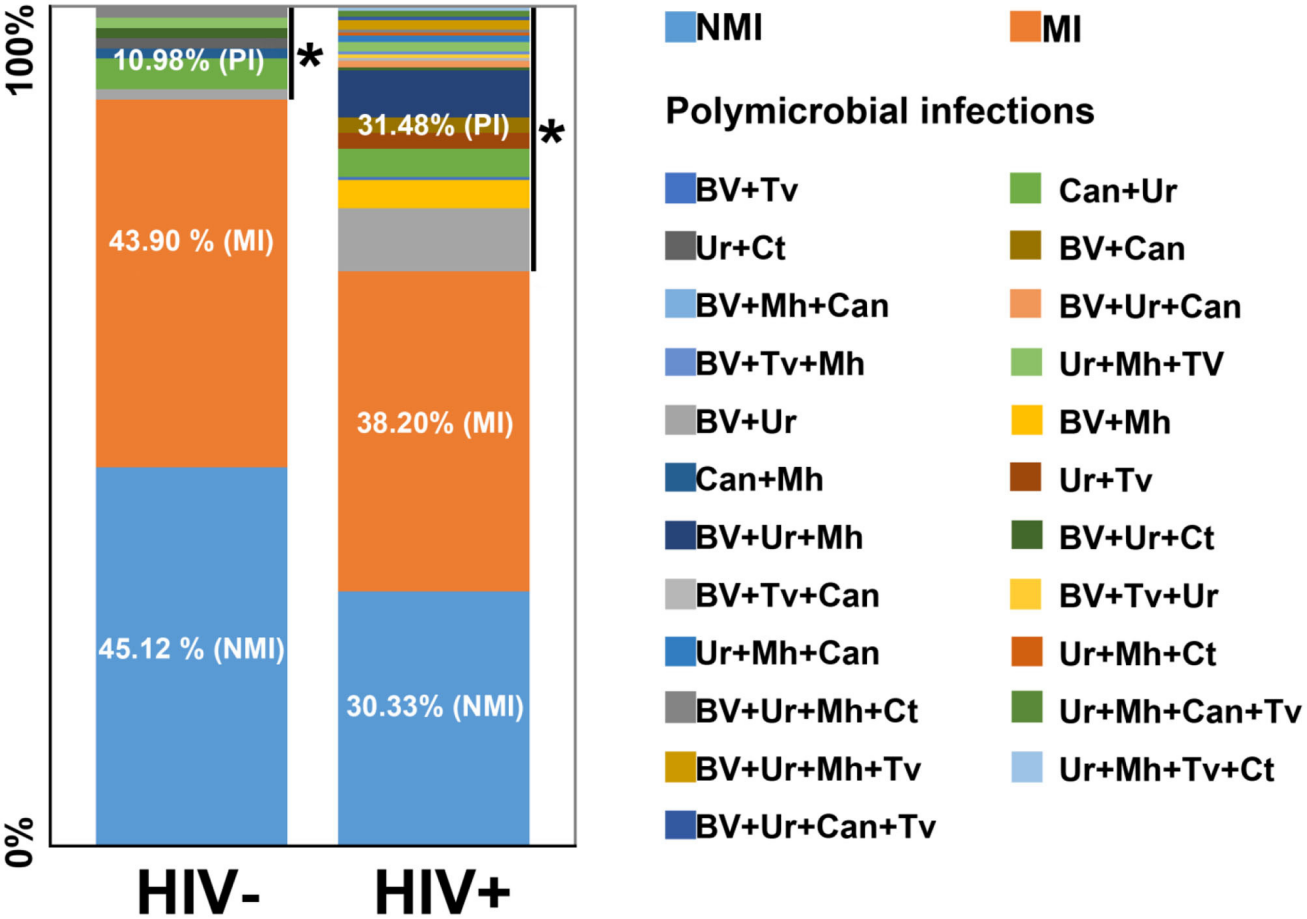
Distribution of infections in both HIV– and HIV+ populations. Tv, *Trichomonas vaginalis*; BV, bacterial vaginosis; Ur, *Ureaplasma* sp; Mh, *Mycoplasma hominis;* Can, Candidiasis; Ct, *Chlamydia trachomatis*; MI, monomicrobial infection; NMI, no microbial infection; PI, polymicrobial infection. *Statistically significant (*p* < 0.05). OR: 3.85, confidence interval 1.75–8.5.

Both analyzed (HIV– and HIV+) populations were subdivided into three different subgroups: those with NMI (HIV– = 45.12% and HIV+ = 30.33%), with MI (HIV– = 43.9% and HIV+ = 38.20%) and with PI (HIV– = 10.98% and HIV+ = 31.48%). This last type was three times more common in HIV+ than in HIV– subjects (^*^p = 0.0004; Figure 1). In HIV+ subjects, the more common combination was BV plus *Ureaplasma* sp. (*n* = 20), while candidiasis plus *Ureaplasma* sp. was more common in HIV– subjects (Figure 1).

### Socio-Demographic-Related ITS Issues

Most of the women included in this study were monogamous (HIV– = 86.6%, HIV+ = 84.8%) and reported two or three sexual partners in their sexual lives (HIV– = 41.5%, HIV+ = 43%). Higher levels of education were observed in HIV– subjects (college = 67.9%), while high school was the most common level for HIV+ subjects (43.7%). In HIV– patients, age (*p* = 0.690ç), condom use (*p* = 0.727), beginning of sexual life (*p* = 0.923ç), total sexual partners (*p* = 0.181ç), current sexual partners (*p* = 0.862ç) and level of education (*p* = 0.877ç) did not have significant relationships with the possibility of acquiring an infection. Both groups had a similarly low frequency of condom use (HIV– = 31.7%, HIV+ = 30.6%). However, in the HIV+ patients (Table 2), condom use was significantly associated with the possibility of acquiring another genital infection (both BV and STIs) (only HIV+ = 40.3% vs. BV or STI plus HIV+ = 26.5%; ^*^p = 0.028).

**Table 2:** Main variables affecting infection frequency among HIV+ subjects.

**Variable**	**Group**	**NMI (%)**	**Infected (%)**	**Total**	***P*-value**
Age	18–20	3 (3.7)	7 (3.8)	10 (3.7)	0.203
	21–25	4 (4.9)	19 (10.2)	23 (8.6)	
	26–30	9 (11.1)	31 (16.7)	40 (15.0)	
	31–35	16 (19.8)	46 (24.7)	62 (23.2)	
	36–40	25 (30.9)	45 (24.2)	70 (26.2)	
	41–45	21 (25.9)	28 (15.1)	49 (18.4)	
	46–50	3 (3.7)	10 (5.4)	13 (4.9)	
Total		81 (100.0)	186 (100.0)	267 (100.0)	
Condom use	No	46 (59.7)	133 (73.5)	179 (69.4)	0.028^*^
	Yes	31 (40.3)	48 (26.5)	79 (30.6)	
Total		77 (100.0)	181 (100.0)	258 (100.0)	
Age at first sexual intercourse	≤ 15	26 (32.1)	52 (28.3)	78 (29.4)	0.543
	16–18	29 (35.8)	83 (45.1)	112 (42.3)	
	19–25	23 (28.4)	42 (22.8)	65 (24.5)	
	≥26	3 (3.7)	7 (3.8)	10 (3.8)	
Total		81 (100.0)	184 (100.0)	265 (100.0)	
Number of current sexual partners	0	8 (12.1)	13 (7.9)	21 (9.1)	0.312
	1	56 (84.8)	139 (84.8)	195 (84.8)	
	≥2	2 (3.0)	12 (7.3)	14 (6.1)	
Total		66 (100.0)	164 (100.0)	230 (100.0)	
Number of sexual partners	1	14 (17.7)	31 (17.3)	45 (17.4)	0.431
	2 or 3	38 (48.1)	73 (40.8)	111 (43.0)	
	4 or 5	13 (16.5)	42 (23.5)	55 (21.3)	
	6 to 9	9 (11.4)	14 (7.8)	23 (8.9)	
	≥10	5 (6.3)	19 (10.6)	24 (9.3)	
Total		79 (100.0)	179 (100.0)	258 (100.0)	
Level of education	Any	1 (1.3)	0 (0)	1 (4)	0.664Ç
	Primary	17 (21.3)	49 (27.1)	66 (25.3)	
	High school	35 (43.8)	79 (43.6)	114 (43.7)	
	Bachelor's degree	20 (25)	39 (21.5)	59 (22.6)	
	Higher education	2 (2.5)	4 (2.2)	6 (2.3)	
	Post-graduate	5 (6.3)	10 (5.5)	15 (5.7)	
Total		80 (100.0)	181 (100.0)	261 (100.0)	

* *Statistical significance (p < 0.05); Ç, Fisher's exact test. Since some participants did not answer all the questions, the total is different for some variables. NMI, without any other genital infection, Infected, patients with other genital infections in addition to HIV*.

## Discussion

Comparative studies of different populations could help us to understand and identify the factors causing the increases in number of these infections. Thus, the data generated herein will be a useful complement to the existing surveillance programmes in Mexico, mainly focused on HIV infection but not on other frequent genital disorders.

Research worldwide has recognized BV as one of the most common types of dysbiosis. The results of this study are in agreement with the findings previously reported by Bilardi et al. (22), who stated that the frequencies of BV in undeveloped countries are between 10 and 30%. These frequencies are even greater in HIV+ females, ranging from 35 to 55% (3, 23). The results for Mexican HIV+ patients presented here are also in agreement with these findings. Because some studies have suggested that BV is consistently associated with an increased risk of HIV infection, the opportune diagnosis of BV is crucial in low- and high-risk patients (24). Studies performed in Mexico City over the last 3 years have shown that the BV frequency is between 10 and 39% in HIV– subjects; however, no studies have reported the BV frequency among HIV+ patients (25–27).

Apalata et al. (28) reported that vulvovaginal candidiasis is significantly more common among HIV-infected women than uninfected women, while our results revealed non-significant differences in frequency (10.97% in HIV– and 11.61% in HIV+ patients). Among HIV+ patients, *Candida albicans* was the most common species, followed by *Candida tropicalis*. *Candida krusei* was detected only in HIV+ patients. The high prevalence of vaginal candidiasis in HIV+ is correlated with the condition of immunosuppression that HIV causes in the patients (3, 29), and our results are in agreement with those of previous reports.

In our results, trichomoniasis was significantly more common in HIV+ (8.23%) than in HIV– (1.21%) subjects (*p* = 0.025). These results indicate that the infected women and their sexual partners did not use condoms frequently; therefore, this STI is an indicator of the sexual behavior of a population and its presence both in our results and in the international literature associated with non-use of condoms (30) (Table 2). Worldwide, among HIV-positive women, the prevalence of TV ranges from 6 to 27% (3); however, no studies have reported trichomoniasis among HIV+ in Mexico.

The *C. trachomatis* frequency detected in HIV– subjects in this study is similar to that reported in previous studies of Mexican populations (HIV– = 6.09%) (16). Although there are insufficient data on Mexican HIV+ patients (HIV+ = 1.82%) to make an accurate comparison, the low frequency of *C. trachomatis* in HIV+ compared with HIV– subjects might have been due to the treatment administered, given the syndromic management of patients who are newly diagnosed as seropositive, which includes the use of broad-spectrum antibiotics (31).

No cases of gonorrhea were detected by culture or nucleic acid amplification technique (NAAT) in HIV– or HIV+ subjects. These results concur with those previously reported for HIV– individuals by Hernández-Martínez et al. (16).

Different risk factors, including insecure sexual behavior, such as not using condoms and sexual intercourse with multiple partners, have been previously associated with *C. trachomatis* and *N. gonorrhoeae* infections (32). In the present study, the unique risk factor identified among the groups was a low frequency of condom use, which might have been the cause of the high detection rates of *C. trachomatis*.

The roles of *Ureaplasma* sp. and *M. hominis* as vaginal pathogens remain controversial. However, their detection in women of child-bearing age is important because of the possibility of horizontal transfer of these bacteria during the delivery of new-borns, who can develop atypical pneumonia. To summarize the information about the possible roles of these bacteria in the genital tract, they were detected using the IST-2 system. A result was considered positive only when the bacteria were present at rates higher than ≥10^4^ CFU. This bacterial concentration has increased correlations with female vaginitis, cervicitis and BV (33).

*Ureaplasma* sp., *M. hominis* and mixed infections (Mh+Ur) in HIV– individuals were similar to those described in previous reports (16). The frequency of Mh+Ur infections was significantly higher in HIV+ individuals than in HIV– individuals (Mh+Ur = 10.86% vs. Mh+Ur = 2.43%, respectively) (0.003^*^). The OR test showed that compared with the HIV– subjects, the HIV+ subjects in our study had a 6.64-fold increased risk of acquiring a mixed infection. The *Ureaplasma* sp. frequency was high in the HIV– subjects, highlighting the importance of routine *Mycoplasma* and *Ureaplasma* detection in clinical laboratory analyses.

The frequencies of polymicrobial infection in HIV+ and HIV– subjects were 31.48 and 10.98%, respectively, which might be attributable to a decrease in normal microbiota bacteria (*Lactobacillus*), which has been previously reported in HIV+ subjects (3, 34). This bacterium possesses numerous potentially beneficial properties, including lactic acid, bacteriocin and H_2_O_2_ production and adherence to vaginal mucosa; thus, it has antagonistic activity toward pathogens (35, 36). These results are in agreement with those of previous studies showing that dysbiosis and polymicrobial infections are more common among HIV+ individuals (24).

Regarding the HIV+ patients in our study, the most common polymicrobial infections were BV and genital mycoplasmas (BV+Ur = 7.49%; BV+M h = 3.37%; and BV+Ur+Mh = 5.61%); in all cases, Mh or Ur showed high concentrations (>10^4^ CFU). These results concur with those of previous reports that state that women with BV not only have *M. hominis* more often but also in much larger numbers (up to 10,000-fold more) than women who do not have BV. Other studies have shown that a large number of *M. hominis* organisms play important roles in BV biofilms, where they act synergistically with *G. vaginalis* and other anaerobic bacterial genera (9, 37). In addition, Sha et al. (38) demonstrated that the level of HIV in the female genital tract is increased in the presence of *M. hominis*. Thus, sex partners of HIV-infected women with BV and *M. hominis* may be at increased risk of acquiring HIV infection.

A survey was conducted to identify risk factors that might be related to the acquisition of genital infections diagnosed in this study. Similar to previous studies on HIV– individuals, we did not find any significant association with the analyzed demographic, behavioral or clinical characteristics of the enrolled participants (i.e., age, age at first sexual intercourse, number of sexual partners in the past, number of current sexual partners, condom use, and level of education) (16, 39). Previous authors have described the limitations of self-reported sexual behavior questionnaires. Audio computer-assisted self-interview has been proposed as an improved method to obtain this kind of sensitive information and reduce possible bias. In our study, it was not possible implement this technological tool. Rather, we improved the data quality by using different previously recommended strategies (40). Our main strategies were to explicitly ensure the confidentiality of the data, use language that was easily understandable, and question the person in charge of sampling to identify inconsistencies between self-reports of sexual behaviors and STI laboratory results.

Some authors have reported that HIV+ patients represent a high-risk population with regard to both behavior and STI co-infections, mainly owing to CD4 immunological alterations (13, 34). The survey results showed that only the use or non-use of condoms influenced the absence or presence of cervicovaginal infections, respectively and was the only variable that presented a significant difference in our study. These findings are similar to those of other reports in the literature, as condom use reduces contact between sexual partners, thereby decreasing the exchange of microbiota and possible sexually transmitted pathogens (39).

In conclusion, our results show that HIV+ status was significantly associated with a high frequency of BV and polymicrobial infections, highlighting the importance of women with HIV attending routine microbiological evaluations to manage genital infections because women with <200 CD4 cells/mm^3^ have more persistent infections and that the presence of certain microorganisms, as *M. hominis* can increase the level of genital HIV by 100 times (13), which is a major concern in this population in which these bacteria are present and condoms are not used consistently. A comprehensive understanding of the rates of these infections in Mexican women is necessary and should be a component of the community-based assessment of STIs and genital infections.

## Data Availability Statement

The raw data supporting the conclusions of this article will be made available by the authors, without undue reservation.

## Ethics Statement

The studies involving human participants were reviewed and approved by the ethics committees Clínica Especializada Condesa (CEC) CECITS0614 and Escuela Nacional de Ciencias Biológicas del Instituto Politécnico Nacional (ENCB-IPN) CEI-ENCB 001/2013. The patients/participants provided their written informed consent to participate in this study.

## Author Contributions

KN-M, VR-G, CS-J, YM-I, and RD-D participated in the sampling and data acquisition. KN-M, CH-C, and MA-A performed the analysis and/or interpretation of the data. MA-A conceived and designed the study. KN-M and MA-A drafted the manuscript. All authors approved the final version of the manuscript.

## Conflict of Interest

The authors declare that the research was conducted in the absence of any commercial or financial relationships that could be construed as a potential conflict of interest.
